# Lactococcus lactis subsp. lactis infection in waterfowl: first confirmation in animals.

**DOI:** 10.3201/eid0705.017519

**Published:** 2001

**Authors:** J Goyache, A I Vela, A Gibello, M M Blanco, V Briones, S González, S Téllez, C Ballesteros, L Domínguez, J F Fernández-Garayzábal

**Affiliations:** Facultat de Veterinaria, Universidad Complutense, Madrid, Spain.

## Abstract

We report the first description, confirmed by bacteriologic and molecular (polymerase chain reaction and pulsed-field gel electrophoresis) analysis, of an infection in animals caused by Lactococcus lactis subsp. lactis, affecting waterfowl.


### *Lactococcus lactis* subsp. *lactis* Infection in Waterfowl: First Confirmation in Animals

**Joaquín Goyache, Ana I. Vela, Alicia Gibello, María M. Blanco, Víctor Briones, Sergio González, Sonia Téllez, Cristina Ballesteros, Lucas Domínguez, and José F. Fernández-Garayzábal**

Facultad de Veterinaria, Universidad Complutense, Madrid, Spain

We report the first description, confirmed by bacteriologic and molecular (polymerase chain reaction and pulsed-field gel electrophoresis) analysis, of an infection in animals caused by *Lactococcus lactis* subsp. *lactis*, affecting waterfowl.

Until recently, members of the genus *Lactococcus* were considered opportunistic pathogens (1,2). They are often misidentified as enterococci or streptococci (3,4), and the difficulties in correctly identifying them have probably hindered elucidation of their clinical significance. However, the number of clinical cases associated with infections by these microorganisms has increased in the last decade in both humans and animals (5-7). *Lactococcus lactis* subsp. *lactis*, *L. piscium*, and *L. garvieae* are recognized as the species with clinical significance for human and veterinary medicine (2,8). In humans, *L. garvieae* and *L. lactis* subsp. *lactis* have been associated with endocarditis (9,10) and have also been isolated from clinical samples of blood, skin lesions, and urine (5,7). In veterinary medicine, *L. garvieae* and *L. piscium* are pathogenic for various fish species (8,11,12), and *L. garvieae* causes mastitis in ruminants (13,14). However, infection by *L. lactis* subsp. *lactis* in animals has not previously been reported. We present the first microbiologic and molecular evidence for infection produced by *L. lactis* subsp. *lactis* in waterfowl.

### The Study

From September to November 1998, a mass die-off was detected among waterfowl in southwestern Spain, affecting >3,000 birds (0.6% of the total waterfowl population in the area). The species most affected were coots (*Fulica atra*) (26.9%), shovelers (*Anas clypeata*) (25.1%), and mallards (*Anas platyrrhynchos*) (13.8%). Overall, 20% of the birds died. Affected birds showed general weakness, evidenced by drooping wings and sluggishness; approximately 50% had respiratory distress. At necropsy, most animals had mild lung congestion; no other lesions were found at postmortem examination.

Samples from the lungs, liver, and spleen of five diseased birds (one mallard, S-15; three shovelers, S-16, S-18, and S-19; and one coot, S-17) were submitted to the Animal Health Department at the School of Veterinary Medicine of Madrid for microbiologic analysis. After 48 hours of incubation at 37ºC, pure cultures of weakly α-hemolytic catalase-negative cocci were obtained on blood agar plates from samples of lung (S-15, S-16, and S-17) and liver and spleen (S-15, S-16, S-17, and S-18). All 11 clinical isolates had an identical biochemical profile, which was identified as *L. lactis* subsp. *lactis* by the Rapid ID 32 Strep system (bioMérieux España, S.A., Madrid).

*L. lactis* subsp. *lactis* and *L. garvieae* are the two species more frequently found in human and animal infections (2). Routine clinical microbiologic diagnosis requires accurate discrimination of the two species, as their similar biochemical reaction patterns may lead to misidentification (2,4). Although physiologic tests, differences in antimicrobial susceptibility, whole-cell protein, and DNA or RNA analysis (4,7,13) have been proposed to distinguish them, some of these techniques are not reliable or may be too time-consuming, limiting their use for routine identification. For these reasons, the clinical isolates were also identified by a polymerase chain reaction (PCR) assay, which has been successfully used to identify many other pathogens (15,16).

Specific primers LLF 5'-GCAATTGCATCACTCAAAGA and LLR 5'-ACAGAGAACTTATAGCTCCC were designed from diagnostic regions of the *L. lactis* subsp. *lactis* 16S rRNA gene sequence (accession number M58837). PCR amplifications were performed in a 100-µL reaction volume containing 150 ng each of the two primers, 1 mM each of deoxynucleoside triphosphate, 1 U of *Taq* DNA polymerase (Biotools, Inc., Madrid, Spain), and 25 ng of template DNA in 1x reaction buffer. The amplification was carried out in a PTC-100 thermal cycler (MJ Research, Inc., Watertown, MA), under the following conditions: initial denaturation at 94ºC for 2 minutes, followed by 30 cycles of denaturation for 1 minute at 92°C, primer annealing for 1.5 minutes at 50°C, primer extension for 2 minutes at 72°C, and a final extension of 5 minutes at 72°C. The following bacterial strains were used to test the specificity of the PCR assay: *L. lactis* subsp. *lactis*, ATCC 19435 and ATTC 11007; *L. garvieae*, NCFB 2155; four clinical isolates of *Lactococcus garvieae* (1336, 1458, 1982, and 4294, isolated from lactococcosis in trout); *L. piscium*, NCFB 2778; *Streptococcus iniae*, ATCC 29187; *Vagococcus fluvialis*, NCFB 2497; and *Enterococcus faecalis*, CECT 481. All the *L. lactis* subsp. *lactis* clinical isolates generated an expected PCR amplification product of 650 bp. No amplification was observed with any other *Lactococcus* species tested, indicating the specificity of the PCR assay (Figure 1). These results confirmed those of the biochemical identification, as well as the utility of this PCR assay for spe-

Address for correspondence: José F. Fernández-Garayzábal, Departamento Patología Animal I (Sanidad Animal), Facultad de Veterinaria, Universidad Complutense, 28040 Madrid, Spain; fax: +34 91 3943908; e-mail: garayzab@eucmax.sim.ucm.es

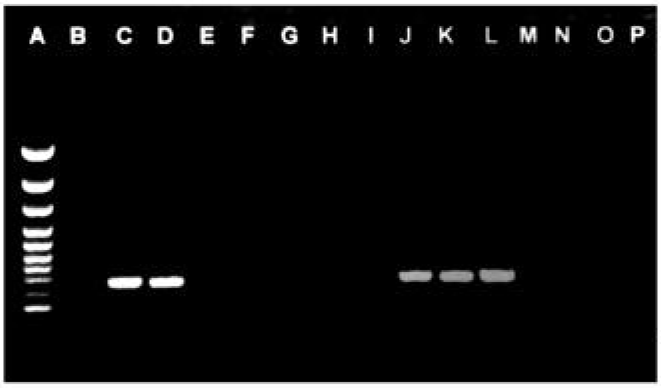

Figure 1. Polymerase chain reaction (PCR) products formed from total DNA of lactococcal strains with primers LLF and LLR. Lane A, molecular size marker; lane B, negative control. *Lactococcus lactis* subsp. *lactis* ATCC 19435 (lane C), *L. lactis* subsp. *lactis* ATCC 11007 (lane D), and the clinical isolates (lanes J, K, and L) generated a PCR amplification product of 650 bp. No amplification was observed from *L. garvieae* NCFB 2155 (lane E), clinical isolates of *L. garvieae* (lanes F, G, H and I), *L. piscium* NCFB 2778 (lane M), *Streptococcus iniae* ATCC 29187 (lane N), *Vagococcus fluvialis* NCFB 2497 (lane O), and *Enterococcus faecalis* CECT 481 (lane P).

cific, rapid, and accurate identification of this microorganism. In addition, the fact that no PCR amplification was observed with clinical isolates when tested with a PCR specific for *L. garvieae* (17) corroborates the identification (data not shown).

Pulsed-field gel electrophoresis (PFGE) with the enzyme *Sma*I was used for molecular characterization of the clinical isolates, as described by Vela et al. (18). This technique has been successfully applied for strain identification and epidemiologic investigations of lactococci (19,20). Indistinguishable restriction patterns were obtained from all clinical isolates (Figure 2), which were clearly distinct from the pulsotypes of *L. lactis* subsp. *lactis* ATCC 19435, *L. garvieae*, and *L. piscium* (data not shown). These PFGE results indicate that infection was produced by a single strain of *L. lactis* subsp. *lactis* in all the animals studied. The bacteriologic and molecular results clearly confirm the isolation of *L. lactis* subsp. *lactis* in waterfowl, the first confirmation of infection in animals caused by this microorganism.

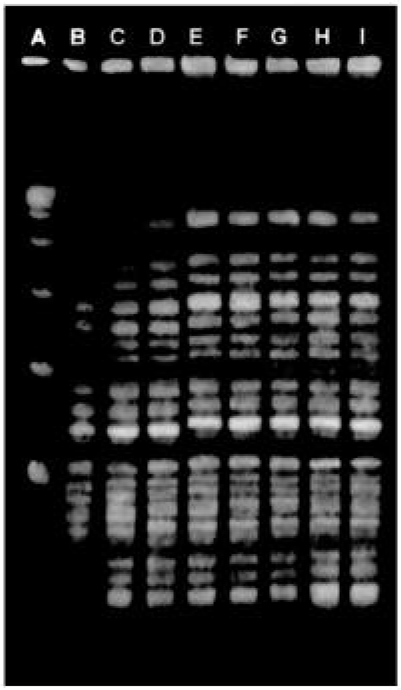

Figure 2. Pulsed-field gel electrophoresis patterns of *Sma*I digests of genomic DNA of *Lactococcus lactis* subsp. *lactis* clinical isolates. Lane A, molecular weight marker; lanes B, D, F, and H, liver isolates from samples S-15, S-16, S-17, and S-18; lanes C, E, and G, lung isolates of samples S-15, S-16, and S-17; and lane I, spleen isolate from sample S-18.

As *L. lactis* subsp. *lactis* is considered nonpathogenic for animals (1,2) and no additional histopathologic or toxicologic studies could be carried out in the diseased animals, we cannot rule out other possible causes for the mass deaths. Therefore, although the PFGE results, together with the recovery of *L. lactis* subsp. *lactis* in pure culture from the clinical samples, may suggest clinical significance, no direct link between the *L. lactis* subsp. *lactis* infection and this episode can be established. Further studies are necessary to elucidate the exact pathogenic potential of this microorganism for waterfowl.

Wild animals, including waterfowl, are known reservoirs for various pathogens (21). We can only speculate about the possibility that waterfowl may be a reservoir for this bacterium. However, wild animal reservoirs for other species of lactococci have been described (22).

Dr. Goyache is associate professor, Facultad de Veterinaria, Universidad Complutense de Madrid. His responsibilities include research and teaching related to clinical microbiology of wildlife and exotic animals.

### References

1. Aguirre M, Collins MD. Lactic acid bacteria and human clinical infection. J Appl Bacteriol 1993;75:95-107.
2. Facklam RR, Elliot JA. Identification, classification, and clinical relevance of catalase-negative, gram-positive cocci, excluding streptococci and enterococci. Clin Microbiol Rev 1995;8:479-95.
3. Schleifer KH. Recent changes in the taxonomy of lactic acid bacteria. FEMS Microbiol Rev 1987;46:201-3.
4. Elliott JA, Facklam RR. Antimicrobial susceptibilities of *Lactococcus lactis* and *Lactococcus garvieae* and a proposed method to discriminate between them. J Clin Microbiol 1996;34:1296-8.
5. Facklam RR, Pigott NE, Collins MD. Identification of *Lactococcus* species from human sources. Proceedings of the XI Lancefield International Symposium on Streptococci and Streptococcal Diseases, Siena, Italy. Stuttgart: Gustav Fischer Verlag; 1990:127.
6. Mannion PT, Rothburn MM. Diagnosis of bacterial endocarditis caused by *Streptococcus lactis* and assisted by immunoblotting of serum antibodies. J Infect 1990;21:317-8.
7. Elliot JA, Collins MD, Pigott NE, Facklam RR. Differentiation of *Lactococcus lactis* and *Lactococcus garviae* from humans by comparison of whole-cell protein patterns. J Clin Microbiol 1991;29:2731-4.
8. Doménech A, Prieta J, Fernández-Garyzábal JF, Collins MD, Jones D, Domínguez L. Phenotypic and phylogenetic evidence for a close relationship between *Lactococcus garvieae* and *Enterococcus seriolicida*. Microbiología SEM 1993;9:63-8.
9. Furutan NP, Breiman RF, Fischer MA, Facklam RR. *Lactococcus garvieae* infection in humans: a cause of prosthetic valve endocarditis [C297]. Proceedings of the 91st America Society of Microbiology Conference. Dallas: American Society of Microbiology; 1991. p. 109.
10. Fefer JJ, Ratzan KR, Sharp SE, Saiz E. *Lactococcus garvieae* endocarditis: report of a case and review of the literature. Diagn Microbiol Infect Dis 1998;32:127-30.
11. Eldar A, Ghittino C, Asanta L, Bozzetta E, Goria M, Prearo M, et al. *Enterococcus seriolicida* is a junior synonym of *Lactococcus garvieae* a causative agent of septicemia and meningoencephalitis in fish. Curr Microbiol 1996;32:85-8.

12. Eldar A, Goria M, Ghittino C, Zlotkin A, Bercovier H. Biodiversity of *Lactococcus garvieae* strains isolated from fish in Europe, Asia, and Australia. Appl Environ Microbiol 1999;65:1005-8.
13. Collins MD, Farrow JAE, Phillips BA, Kandler O. *Streptococcus garvieae* sp. nov. and *Streptococcus plantarum* sp. nov. J Gen Microbiol 1983;129:3427-31.
14. Teixeira LM, Merquior VLC, Vianni MCE, Carvalho MGS, Fracalanzza SEL, Steigerwalt AG, et al. Phenotypic and genotypic characterization of atypical *Lactococcus garvieae* strains isolated from water buffalos with subclinical mastitis and confirmation of *L. garvieae* as a senior subjective synonym of *Enterococcus seriolicida*. Int J Syst Bacteriol 1996;46:664-8.
15. Amann RI, Ludwig W, Schleifer K. Phylogenetic identification and *in situ* detection of individual microbial cells without cultivation. Microbiol Rev 1995;59:143-69.
16. Gibello A, Blanco MM, Moreno M, Cutuli MT, Doménech A, Domínguez L, et al. Development of a PCR assay for detection of *Yersinia ruckeri* in tissues of inoculated and naturally infected trout. Appl Environ Microbiol 1999;65:346-50.
17. Zlotkin A, Eldar A, Ghittino C, Bercovier H. Identification of *Lactococcus garvieae* by PCR. J Clin Microbiol 1998;36:983-5.
18. Vela AI, Vázquez J, Gibello A, Blanco MM, Moreno MA, Liébana P, et al. Phenotypic and genetic characterization of *Lactococcus garvieae* isolated in Spain from lactococcosis outbreaks and comparison with isolates of other countries and sources. J Clin Microbiol 2000;38:3791-5.
19. Tanskanen EI, Tulloch DL, Hillier AJ, Davidson BE. Pulsed-field gel electrophoresis of *Sma*I digests of lactococcal genomic DNA, a novel method of strain identification. Appl Environ Microbiol 1990;56:3105-11.
20. Carvalho MG, Vianni MCE, Elliot JA, Reeves M, Facklam RR, Teixeira LM. Molecular analysis of *Lactococcus garvieae* and *Enterococcus gallinarum* isolated from water buffalos with subclinical mastitis. Adv Exp Med Biol 1997;418:401-4.
21. Hannam DAR. Zoonoses and health implications including COSHH. In: Beynon PH, Forbes NA, Harcourt-Brown NH, editors. Manual of raptors, pigeons and waterfowl. Ames: Iowa State University Press; 1996. p. 113-5.
22. Kusuda R, Salati F. *Enterococcus seriolicida* and *Streptococcus iniae.* In: Woo PTK, Bruno DW, editors. Fish diseases and disorders. Vol 3. Viral, bacterial and fungal infections. Wallingford: CABI Publishing; 1999. p. 303-17.
